# Extramedullary plasmacytoma in the right pulmonary hilum

**DOI:** 10.1590/0100-3984.2015.0074

**Published:** 2015

**Authors:** Lenara Renó Arbex Coelho, Gabriel Pinheiro Coelho, Rodolfo Mendes Queiroz, Marcus Vinicius Nascimento Valentin

**Affiliations:** 1 Documenta - Hospital São Francisco, Ribeirão Preto, SP, Brazil.

*Dear Editor,*

A 53-year-old black, asymptomatic man, driver, being assessed to be released for physical activity. The patient denied smoking as well as having comorbidities.

**Figure 1:**
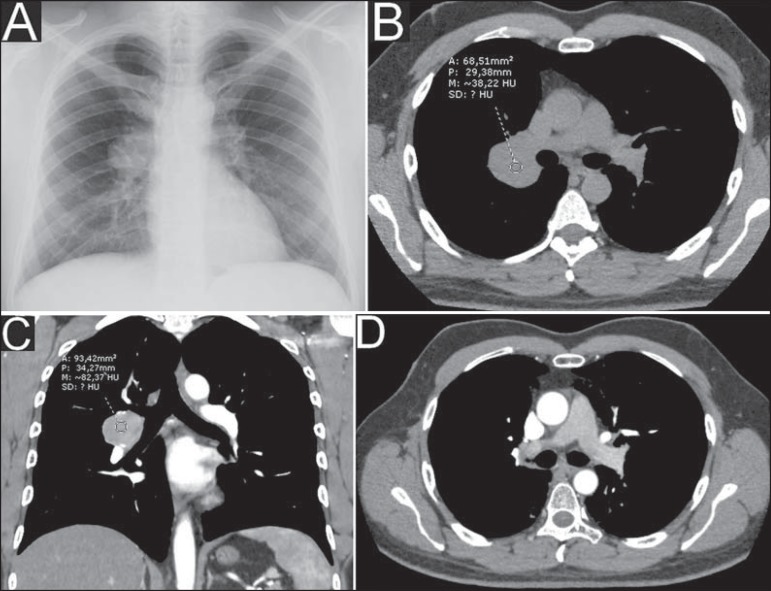
Chest radiography (**A**) showing ovoid opacity in the right hilar region. Axial chest CT section (**B**) at precontrast phase demonstrating circumscribed, round opacity with soft parts attenuation in the hilar region at right, and presenting enhancement after intravenous contrast agent injection, observed at coronal tomographic reconstruction (**C**). Contrast-enhanced axial chest CT section (**D**) after radiotherapy, where the previously described opacity is not characterized anymore, suggesting a good therapeutic response.

Chest radiography performed on February 1st, 2011 showed ovoid opacity in the right hilar region, with no other abnormality (Figure 1A). Chest computed tomography (CT) performed on March 13, 2011 identified circumscribed round opacity with soft parts attenuation in the right hilar region, presenting enhancement after intravenous contrast agent injection, adjacent to the ipsilateral main pulmonary artery and its branches. Absence of other findings (Figures 1B and 1C).

Lesion biopsy result: *macro/microscopy* - hypercellular light-brownish fragments showing well-differentiated plasmacytoid cells with small, eccentric and hyperchromatic nuclei; *immunohistochemical analysis* - positive for CD138 and lambda antibodies; and negative for CD3, CD20, AE1/AE3 and kappa antibodies.

The investigation proceeded with abdominal CT (on May 16, 2011) that showed the presence of a liver cyst and signs of fat infiltration into the liver; normal blood count; negative Bence-Jones proteinuria; protein electrophoresis with no abnormalities; absence of noteworthy findings at bone scintigraphy and bone marrow aspiration.

Radiotherapy was the treatment of choice, with satisfactory response.

Chest CT performed on November 9, 2012 (Figure 1D) and other radiological studies with no suspect finding of disease recurrence/progression until May 20, 2015.

*Diagnosis*: extramedullary plasmacytoma (EMP) in the pulmonary hilum.

Plasmacytoma are primarily classified into solitary bone marrow/bone plasmacytoma (solitary myeloma), extramedullary plasmacytoma or one of multiple myeloma components^(1,2)^. Such tumors are constituted of plasmacytoid cells, presenting malignant degeneration and producing a specific immunoglobulin molecule^(3-7)^.

The incidence of EMP is higher in men than in women, at a 3-4:1 ratio, most frequently occurring around the age of 50-60^(1,4,6,7)^. It is estimated that such tumor represents 2-4% of plasmacytoid neoplasms whose most relevant representative is the multiple myeloma^(1,3-7)^, the latter representing up to 1% of all general malignancies^(8)^.

Approximately 80-90% of EMP cases involve craniocervical structures (upper aerodigestive tract; larynx; nasopharynx; tonsilla; nasal and paranasal cavities)^(1-8)^, but the number of cases does not reach 1% of all neoplastic head and neck lesions^(5)^. Other sites such as gastrointestinal and urogenital tracts, central nervous system, thyroid, parathyroid glands, salivary glands, lymph nodes, skin, lungs, and breasts are uncommon^(2,3,5,6)^. Lymph node involvement in pulmonary hila is extremely rare, with rates as low as less than 2% of cases^(2)^.

Generally, they present as masses with nonspecific soft parts density^(3)^. Histologically, such tumors do not originate directly from the bone marrow and cannot be distinguished from multiple myelomas. Also the differentiation from plasmacytoid cell granulomas and other inflammatory reactions is difficult, essentially requiring immunophenotyping^(1,4)^.

The diagnosis of EMP is made after rigorous investigation to rule out the presence of multiple myeloma, highlighting the histological confirmation by means of immunohistochemical analysis, biopsy/bone marrow puncture showing < 5% of plasmacytoid atypia; to rule out the presence of osteolytic lesions, serum and urinary protein dosage and electrophoresis (to rule out the presence of M and Bence-Jones proteins, respectively); and non-existence of anemia^(1-4,6,7)^.

EMP may be the initial manifestation of multiple myeloma, with progression in about 30% of cases^(1,2,7)^.

Treatments of choice include radiotherapy due the high radiosensitivity in 80-100% of cases, and surgery for localized lesions^(1,3-5,8)^. With such treatments, one observes recurrence and dissemination rates between 20% and 40%^(1^^,,^^2,5-7)^, and ten-year survival in 70% of cases^(1,5-7)^.
